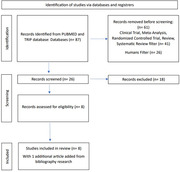# Significance of Donanemab Versus Placebo in Alzheimer’s Disease Pathogenesis. A Systematic Review

**DOI:** 10.1002/alz.085764

**Published:** 2025-01-09

**Authors:** Arid Barragán Ortíz, Marcos Arreola Flores, Christan J Monge Ortega, Sofia Andrade Lara, David A Chávez Castillo, Sergio M Carrillo Rodríguez, Thania F Estrada Ortega, Maria F Estrada Posadas, Gilberto Mauricio Suárez

**Affiliations:** ^1^ Universidad Autónoma del Estado de México, Toluca, EM Mexico; ^2^ Universidad Autónoma de San Luis Potosi, San Luis Potosi, SL Mexico

## Abstract

**Background:**

Alzheimer’s Disease (AD) is a neurodegenerative disease, characterized by a decrease in cognitive and behavioral functions of patients. Between the multiple potential disease‐modifying therapeutics for AD, we have monoclonal antibodies as aducanumab, lecanemab, and donanemab. Recent results from the TRAILBLAZER‐ALZ trial, highlighted donanemab as a promising monoantibodies treatment of early symptomatic AD. Donanemab targets the N‐terminal region of pyroglutamate Aβ plaques. It is designed to bind selectively to these plaques. This targeting mechanism offers a focused approach in the treatment and potential amelioration of the disease’s symptoms.

**Method:**

systematic search in the meta search engines of Pubmed and TRIP database, described in the diagram

**Result:**

Donanemab follows a two‐compartment model with first order elimination and in several studies found that reduction of amyloid plaque is associated with maintenance of donanemab threshold serum concentrations above 4.43 µg/ml. The difference in iADRS score changed ‐6.86 in donanemab group and ‐10.06 in placebo group. Also amyloid plaques clearance measured by florbetapir PET was 85.06 centiloids more than in the placebo group, −84.13 centiloids for donanemab vs. 0.93 centiloids for placebo. In early stages of Alzheimer’s disease, donanemab as treatmenet, resulted in a better composite score for cognition and for the ability to perform activities of daily living than placebo at 76 weeks. Among the side effects that were seen across studies were amyloid‐related edema or stroke (mostly asymptomatic) occurring with donanemab and no exposure threshold was identified as being associated with an increased risk of ARIA‐E. The apparent effect of donanemab on ARIA‐E was generated primarily in participants who were carriers of the APOE ε4 gene.

**Conclusion:**

Preliminary data indicates its potential in effectively impeding the progression of the disease, as observed in the extracted articles. In order to comprehensively grasp the implications of the observed rise in CSF Aβ42 and its correlation with cognitive and clinical effects. While there is limited research available, the current evidence suggests that donanemab is indeed efficacious in halting the advancement of the disease. Donanemab has emerged as a promising option, yet its status is not definitive due to the associated risk of developing ARIA‐E during its administration.